# From genomic medicine to precision medicine: highlights of 2015

**DOI:** 10.1186/s13073-016-0265-4

**Published:** 2016-01-29

**Authors:** Charles Auffray, Timothy Caulfield, Julian L. Griffin, Muin J. Khoury, James R. Lupski, Matthias Schwab

**Affiliations:** European Institute for Systems Biology and Medicine, CNRS-ENS-UCBL, Université de Lyon, 69007 Lyon, France; Faculty of Law and School of Public Health, Health Law Institute, University of Alberta, Alberta, T6G 2HS Canada; Department of Biochemistry, University of Cambridge, Cambridge, CB2 1GA UK; Medical Research Council Human Nutrition Research, Elsie Widdowson Laboratory, 120 Fulbourn Road, Cambridge, CB1 9NL UK; Office of Public Health Genomics, Centers for Disease Control and Prevention, Atlanta, GA 30329 USA; Department of Molecular and Human Genetics, Department of Pediatrics, and Human Genome Sequencing Center, Baylor College of Medicine, One Baylor Plaza Room 604B, Houston, 77030 TX USA; Texas Children’s Hospital, Houston, 77030 TX USA; Dr. Margarete Fischer-Bosch-Institute of Clinical Pharmacology, 70376 Stuttgart, Germany; Department of Clinical Pharmacology, University Hospital, 72076 Tübingen, Germany; Department of Pharmacy and Biochemistry, University of Tübingen, 72076 Tübingen, Germany

2015 has been an exciting year for genomic medicine. We asked our Section Editors to discuss the breakthroughs in their fields of expertise, and what these might mean for the future. As in previous years, exome and whole-genome sequencing are leading the way in our understanding of disease mechanisms, their diagnosis and treatment, while information about the protein products of the genome has also grown, driven by novel technological advances. Precision medicine is now taking off as an important topic in the public health sphere and in education, and no discussion of 2015 would be complete without a mention of the huge advances in gene editing technologies, the implications of which have dominated ethics and policy debate.

## **Clinical genomics for functional annotation of the human genome**

Functional annotation of the human genome through gene discovery took a notable “leap” in 2015 with insight into gene function provided using Mendelian genomics approaches, primarily exome sequencing, to study the specific rare variants underlying disease trait manifestation.

The numbers of gene discovery publications are too numerous to single out any one; however, the UK Wellcome Trust Deciphering Developmental Disorders (WTDDD) [1, 2] and US National Human Genome Research Institute Centers for Mendelian Genomics (NHGRI CMG) [3] initiatives and other such efforts around the world are having a noticeable impact with direct and immediate clinical practice implications. The global impact of this work on human genetics, genomics and the practice of clinical medicine is palpable. Personal genome information and individual variation based on whole exome studies is increasingly utilized to identify a molecular diagnosis and help formulate a differential clinical diagnosis, particularly for diagnostic dilemmas that have evaded an etiologic diagnosis by conventional clinical approaches. *Genome Medicine* dedicated an entire issue (July 2015) to this topic with notable papers describing advances in emergency medical genomes [4] and reviewing the integration of clinical phenotypic information into the analyses of whole exome study data [5].

This effort to functionally annotate the human genome has been greatly facilitated by a web-based platform for rare disease gene discovery—the Matchmaker Exchange [6]—that has enabled the worldwide connection of families with rare diseases and the clinicians caring for them to contribute to this effort for humanity. Such rare variant studies are also beginning to yield insights into complex traits such as scoliosis and neuropathy, and the genetic architecture and genetic models potentially underlying common and complex traits including compound inheritance [7] and mutational burden [8].

As we look forward in 2016 the clinical data available that may enhance biological research efforts will likely continue to explode. More and more we may find that model organism studies look toward human genetics and medical genetics data to provide important insights into biology and in so doing reveal fundamental understanding of disease processes. Moreover, such new understanding may foment novel approaches to disease management and potentially enable therapeutic intervention for rare variant associated disease.

*James R. Lupski,*

*Section Editor,*

*Genomics and epigenomics of disease*

## Implementation of pharmacogenomics and beyond

There is increasing awareness of pharmacogenomics (PGx) as a key component in personalized medicine. A recent evaluation of all 517 medications labeled by the European Medicines Agency (EMA) between 1995 and August 2014 demonstrated that 15 % of these drugs include PGx information which covers pharmacokinetic (for example, drug metabolism/transport) and pharmacodynamic targets [9]. Warnings are related to therapeutic indications, posology and method of administration, and contraindications, thereby directly influencing treatment decisions. Predominantly anticancer drugs are affected by these PGx labels.

Major efforts have been initiated to implement genetic testing of actionable pharmacogenes into clinical practice. The Clinical Pharmacogenetics Implementation Consortium (CPIC), a group of international experts, is working on evidence-based guidelines which are standardized and provide PGx data to individualize the prescribing of drugs [10]. However the clinical implementation process is hampered by several issues such as the reluctance of professional societies, clinicians, and reimbursement organizations. One major concern is the strict request for prospective controlled clinical trials demonstrating state-of-the art evidence for the superiority of an individualized approach for drug prescription based on pharmacogenetic testing. Regarding this issue, a landmark randomized clinical trial has been published corroborating the clinical utility of upfront genetic testing for the thiopurine *S*-methyltransferase gene *TPMT* to avoid hematotoxicity associated with thiopurine drugs, such as azathioprine [11]. Convincing evidence had already been established in the 1990s that patients with decreased thiopurine methyltransferase enzyme activity and standard dosing of thiopurines are at risk of developing myelosuppression. Nevertheless the progress of clinical implementation is still slow and hopefully will derive benefit from this recent trial. Another example is dihydropyrimidine dehydrogenase (DPYD) PGx and drug use of fluoropyrimidines (for example, fluorouracil). Just 30 years after the first description of fatal outcome of fluorouracil administration due to DPYD deficiency, a meta-analysis in 2015 has provided strong evidence that several rare *DPYD* variants contribute to fluoropyrimidine-induced severe toxicity [12]. Although fluoropyrimidines are frequently prescribed, *DPYD* testing is still not officially recommended [10].

The vision of personalized medicine and PGx is rather complex and strategies are needed for better clinical implementation. The use of electronic clinical decision support (eCDS) like the US-initiative eMERGE (Electronic Medical Records and Genomics) will promote the progress of clinical implementation of PGx. However comprehensive and systematic phenotypic characterization of the patient, including diseases and drug therapy, by high-throughput technologies is mandatory. Computer-based, direct interview methods are available to assess the patients’ phenotypes independent of physician input in a standardized manner [13]. Multi-omics data in addition to genomic information which may improve prediction of drug response will be routinely available in the near future. Thus systems-based approaches and drug-specific algorithms are required, a concept which is beyond eCDS tools. The translational, multidisciplinary, and facilitative role of clinical pharmacology may help to achieve this goal [14].

*Matthias Schwab,*

*Section Editor,*

*Pharmacogenomics and personalized medicine*

## The beginning of precision medicine for population health

In 2015, the USA launched the precision medicine initiative that includes two components: one focusing on cancer genomics and treatment, and one focusing on generating data on long-term health and disease in a national cohort research study of one million or more people [15]. While much of this initiative will take years to develop outputs for use in healthcare, a public health perspective is crucial to ensure the initiative’s success in terms of representativeness, generalizability, implementation, and near-term deployment of already established evidence-based genomic findings to save lives and prevent disease [16].

The field of pathogen genomics continues to expand. Molecular technologies are being integrated into the diagnosis, treatment, and control of infections. For example, rapid metagenomic identification of viral pathogens in clinical samples can be accomplished by real-time nanopore sequencing analysis [17]. The Centers for Disease Control and Prevention (CDC) Advanced Molecular Detection initiative is combining genome technologies with bioinformatics and epidemiology to enhance public health surveillance, investigations, and control of infectious diseases [18]. Pathogen genomics is becoming quite helpful globally; for example, in tracking the Ebola virus epidemic transmission in West Africa [19].

We also saw increasing applications of human genetics into public health programs. While genetics has been part of public health since newborn screening began in the 1960s, genetics is becoming a priority area for prevention and treatment of common chronic diseases such as cancer and heart disease. A special 2015 issue of the journal *Healthcare*, focusing on implementation of public health genomics, presented data and information on a range of ongoing public health activities, including reducing the burden of hereditary breast and ovarian cancer, screening children for familial hypercholesterolemia, and engaging medically underserved populations in family history education [20].

Because of the rapid evolution of genomic medicine, a crucial function in public health genomics is to identify evidence-based genomic applications that can improve health, inform and engage various stakeholders, and integrate genomics into ongoing public health programs [21]. To help with this daunting task, the CDC launched in 2015 the Public Health Genomics Knowledge Base in an effort to continue capturing evolving snapshots of the field while tracking the translational trajectory of genome-based discoveries into population health impact [22].

While the precision medicine initiative will take years to mature and lead to tangible discoveries and products, 2016 promises to be an exciting year for the intersection of precision medicine and population health. Some key areas to watch include: (1) developing more robust approaches to obtain empirical data on the impact of genomics and precision medicine on population health; (2) developing metrics for best practices on genomics and population health including indicators for successful implementation and outcomes; and (3) exploring key concepts for the development of “precision public health” [23] beyond genomics, to include a variety of personal and environmental data for preventing disease and promoting health.

*Muin J. Khoury,*

*Section Editor,*

*Genomic epidemiology and public health genomics*

## The view from the proteome, metabolome, and lipidome: life at the end of the central dogma

I have chosen four articles published in 2015 that I feel either demonstrate the versatility of proteomic, metabolomic, or lipidomic approaches to medicine, or will have significant impacts as new approaches in the near future.

While liquid chromatography mass spectrometry (LC-MS) dominates as a tool for both metabolomics and proteomics, users have to decide whether they will perform nontargeted, discovery approaches, which are at best semiquantitative in nature, or targeted approaches relying on triple quadrupole MS, which, while quantitative, only target a limited number of metabolites or peptides. Developments in 2015 have questioned these separate workflows. Guo and colleagues [24] describe an approach, termed sequential window acquisition of all theoretical fragment ion spectra (SWATH)-MS, to acquire the parent ion and fragmentation data of peptides produced from a tissue biopsy by varying the precursor isolation window in a sequential manner. Mass transitions associated with individual peptides are reconstructed in silico and produce spectra comparable to that acquired with a triple quadrupole in terms of specificity but with the global nature of a nontargeted approach. They illustrate this by detecting over 2000 proteins in biopsy samples from patients with renal cell carcinoma.

Similar approaches can be used to reconstruct lipidomes, using the fact that all lipid species consist of a series of building blocks such as individual fatty acids and head groups. Ni and coworkers [25] describe a novel application to identify carbonylated lipid peroxidation products, which may be useful as biomarkers of a range of diseases including atherosclerosis, fatty liver disease, and reperfusion injury, using LipidXplorer software.

One problem with new bioinformatic tools that limits their uptake is that they may be written in a variety of languages. Giacomoni and coworkers [26] borrow an approach from genomics to develop a web-based workflow for LC-MS-based metabolomics that links software from a variety of sources. Workflow4Metabolomics provides a virtual research environment built upon the Galaxy environment [27] linking together open source tools that allow users to completely process their data without extensive experience of the software or tools.

Metabolomics, proteomics, and lipidomics are also delivering in terms of translational medicine. For example, Eiden and colleagues [28] used LC-MS-based lipidomics to identify biomarkers associated with a failure of adipose tissue function in lipodystrophic patients. They identified a characteristic alteration in triglyceride profiles caused by increased de novo lipogenesis in the liver as a consequence of ectopic fat deposition which may be applicable to identifying those with fatty liver in the general population. With new developments in tools and software we should see the range of these metabolomic, proteomic, and lipidomic biomarkers expand and make it into the clinic.

*Julian L. Griffin,*

*Section Editor,*

*Proteomics & metabolomics in medicine*

## Big data and genomic medicine converge to support personalized medicine globally

2015 has witnessed an acceleration of the convergence of big data and genomic medicine that supports the implementation of personalized medicine using systems biology approaches. These are now being developed globally through advanced education programs targeted at research and healthcare professionals as well as the public.

These trends were discussed at the Big Data and Healthy Living Technologies Roundtable organized by BioHealth Computing and the European Scientific Institute, the training branch of CERN, in Archamps, France [29], and during the Big Data in Health Care—Challenges, Innovations and Implementation symposium organized by the Luxembourg Centre for Systems Medicine in Munsbach, Luxembourg [30]. On these two occasions, it became clear that, firstly, the real-life implementation of systems medicine to address important unmet healthcare needs requires novel curricula for the training of the new generation of scientists and medical doctors, and, secondly, that decades of expertise developed in other fields such as particle physics are available to support the development of open standards for the description and exchange of the variety of big data in health.

In relation to the first issue, Siwo et al. highlighted [31] how cloud-computing and genetic testing are being combined in structured education programs to leverage the large genetic variation existing in Africa, thus bringing the continent to the forefront to explain genotype–phenotype–disease relationships. The participants from academic, industrial and governmental organizations also recognized that the current inability to deal with the second widely debated issue, as discussed by Horvitz and Mulligan [32], may constitute a major roadblock in the development of personalized medicine internationally. Indeed, the diversity of legal and regulatory frameworks and the invalidation by the European Court of Justice of the United States Safe Harbor provisions for data transfer across the Atlantic [33] are introducing a high level of uncertainty. In this context, the introduction by Bahr and Schlünder [34] of a code of practice for the secondary use of medical data in scientific research projects, and its consideration in the ongoing revision of the European Directive on Personal Data Protection, may provide a path toward harmonization of regulatory practices.

Through the consolidation of these trends, 2016 should therefore see more advances toward the actual implementation of participatory, preventive, predictive, personalized (P4) systems medicine to understand wellness and tackle unmet medical needs worldwide.

*Charles Auffray,*

*Section Editor,*

*Systems medicine and informatics*

## Gene editing dominates science policy debates

While many genomic policy issues continue to attract significant attention from both the academic community and the general public, the social and ethical challenges associated with the new gene-editing technology dominated in 2015.

The prospect of being able to efficiently edit the human genome—through the application of a technology known as CRISPR-Cas9—has stirred debate about if and when the modification of the human germ line can ever be ethically justified. Some in the ethics community have called for a complete ban, fearing, inter alia, the unknown future consequences of altering the human genome. Those who support the cautious clinical application of gene editing note the potential to cure rare monogenic diseases [35]. The debate culminated in an international scientific summit held in Washington DC at the National Academy of Sciences, where it was suggested that research should continue but—at least for now—a clinical application should not be attempted until governance, safety, and efficacy issues are resolved [36]. This was, in effect, a call for a voluntary moratorium on edits that could be inherited but also support for the research to continue. This conclusion fits with recommendations made by other entities, such as the International Society for Stem Cell Research and the Hinxton Group [37]. It should be noted that in some jurisdictions there are already laws in place that would have an impact on the clinical application of the emerging gene-editing technology. In my home country of Canada, for example, any procedure that results in the alteration of the germ line, even if done to cure a disease like Tay Sachs or Huntington’s, could be categorized as a criminal offence as per the relevant federal law [38]. Given the ongoing debate about the potential scientific and health benefits, the Canadian situation highlights the potential policy dilemmas created by the use of rigid legislative frameworks to regulate unpredictable areas of science.

Another policy challenge that received attention in 2015 was the regulation of direct-to-consumer (DTC) genetic testing. In 2013, the California company 23andMe was prohibited by the US Food and Drug Administration from providing health-oriented testing to its consumers. But in 2015, the company was granted permission to start providing a limited array of services (for example, providing information about “carrier status” for conditions like sickle-cell anemia and cystic fibrosis) [39], thus once again prompting questions about the potential health value (if any) and risks (if any) of these kinds of DTC services.

2016 will undoubtedly see the emergence of new ethical challenges, such as the regulation of the noninvasive prenatal testing market and the growing (and largely evidence-free) DTC genetic testing industry for lifestyle, fitness, and nutrition. But as the science continues to move forward quickly, gene editing seems likely to remain a dominant policy issue in 2016.

*Timothy Caulfield,*

*Section Editor,*

*Ethical, legal and social issues*

## Abbreviations

CDC: Centers for Disease Control and Prevention
DTC: Direct-to-consumer
DPYD: Dihydropyrimidine dehydrogenase
eCDS: Electronic clinical decision support
LC-MS: Liquid chromatography mass spectrometry
PGx: Pharmacogenomics
